# Cancer-specific senescence signature promotes malignant phenotypes and immunotherapy resistance in colorectal cancer

**DOI:** 10.3389/fimmu.2025.1603787

**Published:** 2025-07-24

**Authors:** Wei Wang, Fengyu Ling, Dong Huang, Guomin Luo, Bixia Duan

**Affiliations:** ^1^ Department of Oncology, The Affiliated Yongchuan Hospital of Chongqing Medical University, Chongqing, China; ^2^ Chongqing Municipality Clinical Research Center for Geriatric Diseases (YongChuan Hospital, Chongqing Medical University), Chongqing, China; ^3^ Department of Oncology and Hematology, Fengdu General Hospital, Chongqing, China

**Keywords:** colorectal cancer, immunotherapy resistance, senescence, prognosis, tumor immunity

## Abstract

**Background:**

While cellular senescence in colorectal cancer (CRC) exhibits strong correlations with immunotherapy response and clinical prognosis, its mechanistic basis remains elusive, and validated predictive biomarkers are currently unavailable.

**Methods:**

In this study, we integrated single-cell and bulk transcriptomic data to establish a cancer-specific senescence signature (CSS). Systematic biological characterization revealed that the CSS remodels the tumor microenvironment (TME), primarily through perturbed immune cell infiltration and CD8^+^ T-cell dysfunction. Functional validation via shRNA-mediated CD24 knockdown in HCT116 cells was corroborated by Western blot and flow cytometry. CD24 ablation’s effects on malignant phenotypes were assessed using colony formation, Transwell invasion, wound healing, and proliferation/apoptosis assays (Ki67/Annexin V/TUNEL). CSS-mediated CD8+ T-cell regulation was investigated using palbociclib-induced senescence models (HCT116/SW480). Potential senescence-targeting compounds were identified via the Cancer Therapeutics Response Portal (CTRP) and PRISM databases.

**Results:**

Our analyses validated the CSS as both a prognostic biomarker and immunotherapy predictor in CRC. CSS-high tumors displayed diminished cytotoxic T-cell infiltration and impaired CD8^+^ effector functions (reduced IFN-γ/granzyme B production), while CSS-low tumors showed enhanced T-cell activity. Mechanistic investigations revealed CSS-mediated immunosuppression via MHC class I dysregulation, compromising tumor antigen recognition. Genetic CD24 inhibition suppressed proliferation, migration/invasion and triggered apoptosis. Computational screening identified afatinib as a potent CSS-targeting agent, with *in vitro* studies confirming selective senescent cell growth inhibition through proliferation blockage and apoptosis induction. Notably, CSS-high status predicted immunotherapy resistance.

**Conclusion:**

Collectively, CSS drives tumor aggressiveness and independently predicts unfavorable survival outcomes and immunotherapy resistance in CRC. Notably, afatinib targeting of CSS selectively eliminated senescent cells via apoptosis while inhibiting tumor growth, highlighting its therapeutic potential for CSS-high malignancies.

## Introduction

Colorectal cancer (CRC) is among the most common malignancies worldwide and a major cause of cancer-related mortality. Despite recent progress in surgical resection and adjuvant therapies, which have enhanced clinical outcomes, overall survival rates remain unsatisfactory, as many patients still develop tumor recurrence and distant metastases. Immunotherapy has emerged as a promising treatment strategy for CRC; however, its effectiveness is often limited by tumor heterogeneity and the lack of reliable predictive biomarkers to identify patients likely to respond (1, 2).

Cellular senescence in neoplastic cells, characterized by irreversible cell-cycle arrest and the development of a distinct senescence-associated secretory phenotype (SASP), has emerged as a critical regulator of tumorigenesis and cancer progression. Senescent tumor cells secrete diverse pro-inflammatory cytokines, chemokines, and growth factors, collectively known as the SASP. These factors contribute to tumor microenvironment (TME) remodeling, promote sustained angiogenesis, and facilitate immune evasion mechanisms (3–5). Emerging evidence underscores a critical association between tumor cell senescence and immunotherapeutic response, while also revealing its prognostic influence on overall survival (OS) and progression-free survival (PFS) across multiple malignancies, including CRC (6–8). However, the precise molecular mechanisms governing this relationship remain incompletely understood, and no clinically validated biomarker signature currently exists to reliably predict patient outcomes or therapeutic responses. Given this unmet need, the development of a colorectal cancer-specific senescence signature (CSS) capable of prognostication and immunotherapy response prediction holds substantial translational value. Such a signature would enable risk stratification of patients into clinically relevant subgroups, thereby facilitating personalized therapeutic decision-making and optimized treatment allocation (9–11). Moreover, a deeper mechanistic understanding of how the CSS influences immunotherapeutic efficacy may provide novel conceptual frameworks for developing next-generation immunotherapies. Recent technological breakthroughs, particularly advancements in single-cell RNA sequencing (scRNA-seq) and bulk transcriptomic profiling, have revolutionized our ability to decipher tumor heterogeneity and characterize bidirectional tumor-immune interactions within the TME (12, 13). Machine learning-based prognostic models have also shown great potential in predicting patient outcomes and treatment responses in various cancers (14, 15). These technologies and approaches could be instrumental in identifying a CSS and elucidating its role in CRC progression and immune therapy response.

**Figure 1:**
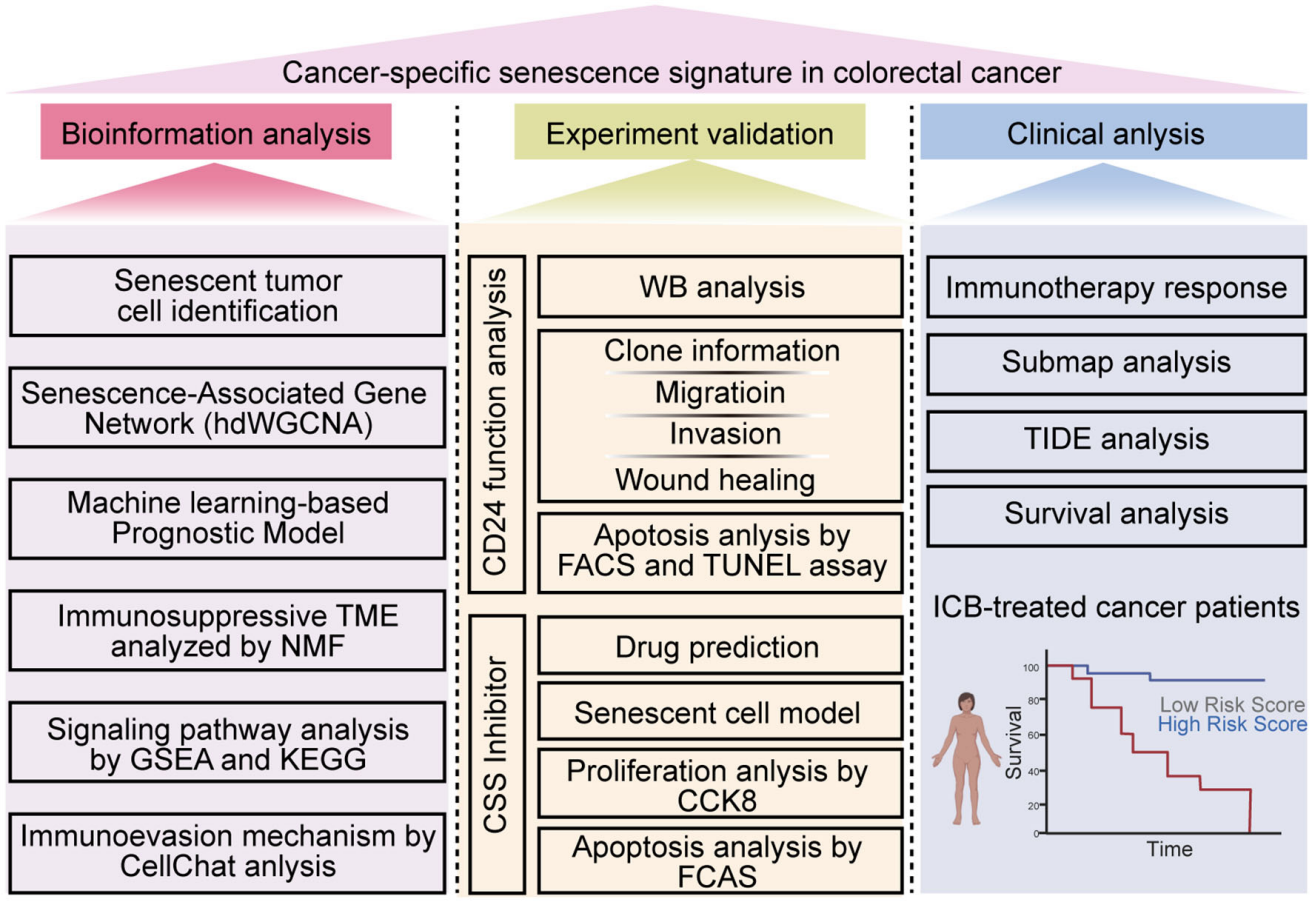
Flow diagram in this study. This study aims to explore the CSS in colorectal cancer through a comprehensive multi-faceted approach. Initially, bioinformatics analysis is conducted, which encompasses identifying senescent tumor cells, constructing a Senescence-Associated Gene Network using hdWGCNA, developing a machine learning-based prognostic model, analyzing the immunosuppressive TME with NMF, conducting signaling pathway analysis via GSEA and KEGG, and investigating immunoevasion mechanisms using CellChat analysis. Subsequently, experimental validation is performed, including WB analysis, assessing CD24 function, evaluating clone information, migration, invasion, and wound healing capabilities, analyzing apoptosis through FACS and TUNEL assays, and utilizing CSS inhibitors for drug prediction, establishing senescent cell models, and conducting proliferation and apoptosis analyses with CCK8 and FCAS. Finally, clinical analysis is carried out, focusing on immunotherapy response, submap analysis, TIDE analysis, and survival analysis in ICB-treated cancer patients. The study provides a detailed workflow to uncover the senescence signature in colorectal cancer, offering insights into potential therapeutic targets and prognostic markers. The image was created by biorender.com (Agreement number: OO28IFS3KR).

In this study, we employed scRNA-seq analysis, transcriptomic profiling, and machine learning-based prognostic modeling to identify a colorectal CSS in CRC. Furthermore, we systematically investigated the influence of CSS on T-cell infiltration and CD8^+^ T cell functionality within the TME, as well as the underlying molecular mechanisms governing CSS-mediated regulation of CD8^+^ T cell activation and effector functions. Finally, through computational drug screening, we aimed to identify potential therapeutic agents capable of targeting and suppressing CSS, which may offer novel treatment avenues for CRC patients (**Figure 1**).

## Methods

### Cell culture

SW480 and HCT116 human colorectal cancer cells were cultured in DMEM medium supplemented with 10% fetal bovine serum (FBS) and 1% penicillin-streptomycin. Cells were maintained in a humidified incubator at 37°C with 5% CO_2_. For experiments, cells were passaged at 70-80% confluence using 0.25% trypsin-EDTA. Stable cell lines were generated by transducing cells with lentivirus particles containing shRNA targeting CD24 or non-targeting control shRNA. Puromycin (2 μg/mL) was used for selection.

### Lentiviral-mediated shRNA knockdown

To achieve stable CD24 knockdown in HCT116 cells, a short hairpin RNA (shRNA)-mediated lentiviral approach was employed. A 65-base pair (bp) DNA oligomer harboring a 19-bp CD24-targeting sequence (5’-AGGCCAAGAAACGTCTTCT-3’) was cloned into the pLVX-Puro vector (GeneCreate, Wuhan, China). Potential off-target effects were assessed using NCBI BLAST, confirming the absence of unintended homologous sequences. As a negative control, a recombinant vector encoding a shRNA targeting firefly luciferase was transduced into parallel cells. Lentiviral particles were generated by transient co-transfection of HEK-293T packaging cells with the pLVX-shRNA1 plasmid, the second-generation packaging plasmid psPAX2, and the phCMV-VSV-G envelope plasmid encoding the vesicular stomatitis virus glycoprotein (VSV-G). Cell-free viral supernatants were harvested 24 h post-transfection, filtered through a 0.45 μm membrane, and cryopreserved at −80°C. For transduction, HCT116 cells were seeded at a density of 5 × 10^4^ cells per well in 0.5 mL culture medium within 6-well plates. Following viral supernatant addition, the medium was replaced after 24 h, and puromycin selection (1 μg/mL) was initiated 48 h post-transduction. Antibiotic selection was maintained for ≥7 days to establish stable knockdown populations. Knockdown efficiency was validated by Western blot analysis, demonstrating significant reduction in CD24 protein expression.

### Western blotting

Protein lysates were prepared using RIPA buffer supplemented with protease inhibitor cocktail. Protein concentrations were quantified by the bicinchoninic acid (BCA) assay following the manufacturer’s protocol. Equal amounts of protein (30 μg per lane) were resolved by 10-12% SDS-PAGE and subsequently transferred to polyvinylidene difluoride (PVDF) membranes. Membranes were blocked with 5% non-fat dry milk in Tris-buffered saline containing 0.1% Tween-20 (TBST) for 1 hour at room temperature, followed by overnight incubation at 4°C with the following primary antibodies: anti-CD24 (1:1000 dilution) and anti-β-Actin (1:2000 dilution). After extensive washing with TBST, membranes were incubated with appropriate horseradish peroxidase (HRP)-conjugated secondary antibodies (1:5000 dilution) for 1 hour at room temperature. Protein bands were visualized using enhanced chemiluminescence (ECL) detection reagents. Band intensities were quantified by densitometric analysis using ImageJ software (National Institutes of Health, Bethesda, MD, USA), with β-Actin serving as the loading control for normalization.

### Colony formation assay

Colony formation was assessed to evaluate clonogenic potential. Cells (500 per well) were seeded in 6-well plates and cultured for 14 days. Medium was refreshed every 3 days. Cells were fixed with 4% paraformaldehyde and stained with 0.1% crystal violet. Colonies (>50 cells) were counted manually. Each experiment was performed in triplicate.

### Transwell migration assay

Cell migration was evaluated using Transwell inserts (8 μm pores). Serum-starved cells (5×10^4/mL) were added to the upper chamber, and 10% FBS was placed in the lower chamber. After 24 hours, migrated cells were fixed, stained, and counted in five random fields. Experiments were performed in triplicate.

### Transwell invasion assay

Invasion was assessed using Matrigel-coated Transwell inserts (8 μm). Serum-starved cells (1×10^5^) were seeded in the upper chamber in serum-free medium, with 10% FBS as chemoattractant in the lower chamber. After 48 h, non-invading cells were removed, and invaded cells were fixed (4% PFA), stained (0.1% crystal violet), and counted in five random fields per insert. All experiments were performed in triplicate.

### Wound healing assay

Wound healing was used to evaluate migration. Cells were seeded in 6-well plates and grown to 90% confluence. A pipette tip created a straight wound, and cells were cultured in serum-free medium. Images were captured at 0 and 24 h, and the migration ratio was calculated using ImageJ.

### Annexin V/PI apoptosis assay

Apoptosis was analyzed using Annexin V-FITC/PI staining. Cells were harvested, washed, and resuspended in binding buffer. Annexin V-FITC (5 μL) and PI (5 μL) were added, and cells were analyzed by flow cytometry. Data were gated to exclude debris, and apoptotic cells were quantified.

### TUNEL assay

Apoptotic cells were detected using the TUNEL assay. Cells were fixed, permeabilized, and incubated with TUNEL reaction mixture for 1 h. Nuclei were stained with DAPI. Fluorescent images were captured, and TUNEL-positive cells were quantified by counting ≥200 cells per field. Experiments were performed in triplicate.

### Flow cytometry analysis

Cells were washed with PBS, stained with fluorochrome-conjugated antibodies targeting CD3, CD8, and CD45, then stimulated with PMA (50 ng/mL) and ionomycin (1 μg/mL) for 4 hours prior to flow cytometry. Detection of surface markers and intracellular cytokines enabled quantitative lymphocyte subset analysis, proliferation assessment (via ki67), and apoptosis measurement (Annexin V/PI) (16).

### CCK8 assay

Colorectal cancer cells (HCT116, SW480) were maintained in RPMI-1640 with 10% FBS and 1% penicillin-streptomycin. After 48-hour afatinib treatment, cell viability was assessed using CCK-8: 10 µL reagent was added per well, incubated at 37°C for 1 hr, and absorbance at 450 nm was measured.

### Single-cell sequencing analysis

Single-cell RNA sequencing (scRNA-seq) data were analyzed using Seurat (v4.0) in R. Cells were retained after stringent quality control with filtering thresholds of nFeature_RNA > 200 & nFeature_RNA < 5000 and percent.mt < 15. Dimensionality reduction was performed following normalization (LogNormalize) and selection of highly variable genes (FindVariableFeatures, selection.method = “vst”, nfeatures = 2000). Data scaling, principal component analysis (PCA, npcs = 30), and batch correction (Harmony, group.by.vars = “orig.ident”) were applied to improve cluster resolution. Uniform Manifold Approximation and Projection (UMAP) was then used for visualization after cell clustering (FindNeighbors, FindClusters, resolution = 0.3). Differential gene expression analysis was performed to identify cluster-specific markers, followed by functional enrichment analysis (Gene Ontology, KEGG) of significant genes to explore biological pathways in CRC (17, 18).

### hdWGCNA analysis

hdWGCNA, an enhanced version of weighted gene co-expression network analysis, was applied to investigate the scRNA-seq data of CRC, enabling the identification of gene expression modules across single-cell populations. This method integrates spatial information to identify gene co-expression modules in the tumor microenvironment (19, 20). We extracted the gene expression matrix and spatial coordinates from the Seurat object and subsequently constructed a weighted gene co-expression network using the createNetwork function in the hdWGCNA package. Gene co-expression modules were then identified via the identifyModules function to detect functionally related gene groups in CRC. Module eigengenes were calculated using getModuleEigengenes and their spatial patterns were visualized with ggplot2. Pearson correlations between module eigengenes and phenotypic features (e.g., cell type annotations) were computed using the cor function. Finally, we performed functional enrichment analysis (Gene Ontology and KEGG pathways) for each module using clusterProfiler to elucidate their biological relevance.

### Transcriptome analysis

Bulk RNA-seq data from COAD and adjacent normal tissues were obtained from the Cancer Genome Atlas (TCGA) database. The raw sequencing data were processed using the “STAR aligner” to map the reads to the human reference genome. Subsequently, gene expression levels were quantified using the “featureCounts” package, providing a precise measurement of transcript abundance for downstream analysis (21). Differentially expressed genes (DEGs) between tumor and normal tissues were identified using the DESeq2 package in R. The genes with adjusted p-values less than 0.05 and log2 fold-change greater than 1 were considered as DEGs. Functional enrichment analysis, encompassing GO and KEGG pathway analysis, was conducted using the “clusterProfiler” package in R. This analysis aimed to uncover the biological functions and pathways associated with DEGs, providing insights into their potential roles in the studied biological context.

### Construction of prognostic model by machine learning

The DEGs derived from transcriptomic analysis were utilized as input features for constructing a prognostic model through machine learning approaches. Feature selection was performed using LASSO regression to identify the most prognostically relevant genes, optimizing both model accuracy and interpretability for clinical outcome prediction. The final model was established via Cox proportional hazards regression, trained on a designated cohort, and subsequently validated in an independent dataset (22, 23). We evaluated the prognostic model’s performance using the concordance index (C-index) and time-dependent receiver operating characteristic (ROC) curves to quantify its predictive accuracy and temporal discriminative ability. Risk scores were calculated for each patient by combining expression levels of prognostic genes with their Cox regression-derived coefficients, providing a quantitative risk estimate. Using the median risk score as the cutoff, we stratified patients into high-risk and low-risk groups for subsequent survival analysis.

### Cell-cell communication analysis

The gene expression data of different cell populations were extracted from the scRNA-seq data. The ligand-receptor pairs between different cell populations were identified based on the gene expression data and the known ligand-receptor interactions in the literature (24, 25). The potential communication pathways were inferred based on the expression levels of ligands and receptors, and the pathways with significant communication were visualized using network diagrams.

### Drug prediction by CTRP and PRISM

Gene expression profiles of CSS were obtained from TCGA-COAD transcriptomic data, while pharmacological response datasets were extracted from CTRP and PRISM databases. Following normalization and batch correction of the expression data, processed drug response information was used to construct a drug-gene interaction matrix. Spearman correlation analysis was performed to assess associations between CSS gene expression patterns and drug response profiles. Compounds demonstrating statistically significant correlations (*P* < 0.05) were identified as potential CSS-targeting therapeutic candidates.

## Results

### Identification of senescence-associated tumor cell subpopulations by scRNA-seq analysis

**Figure 2:**
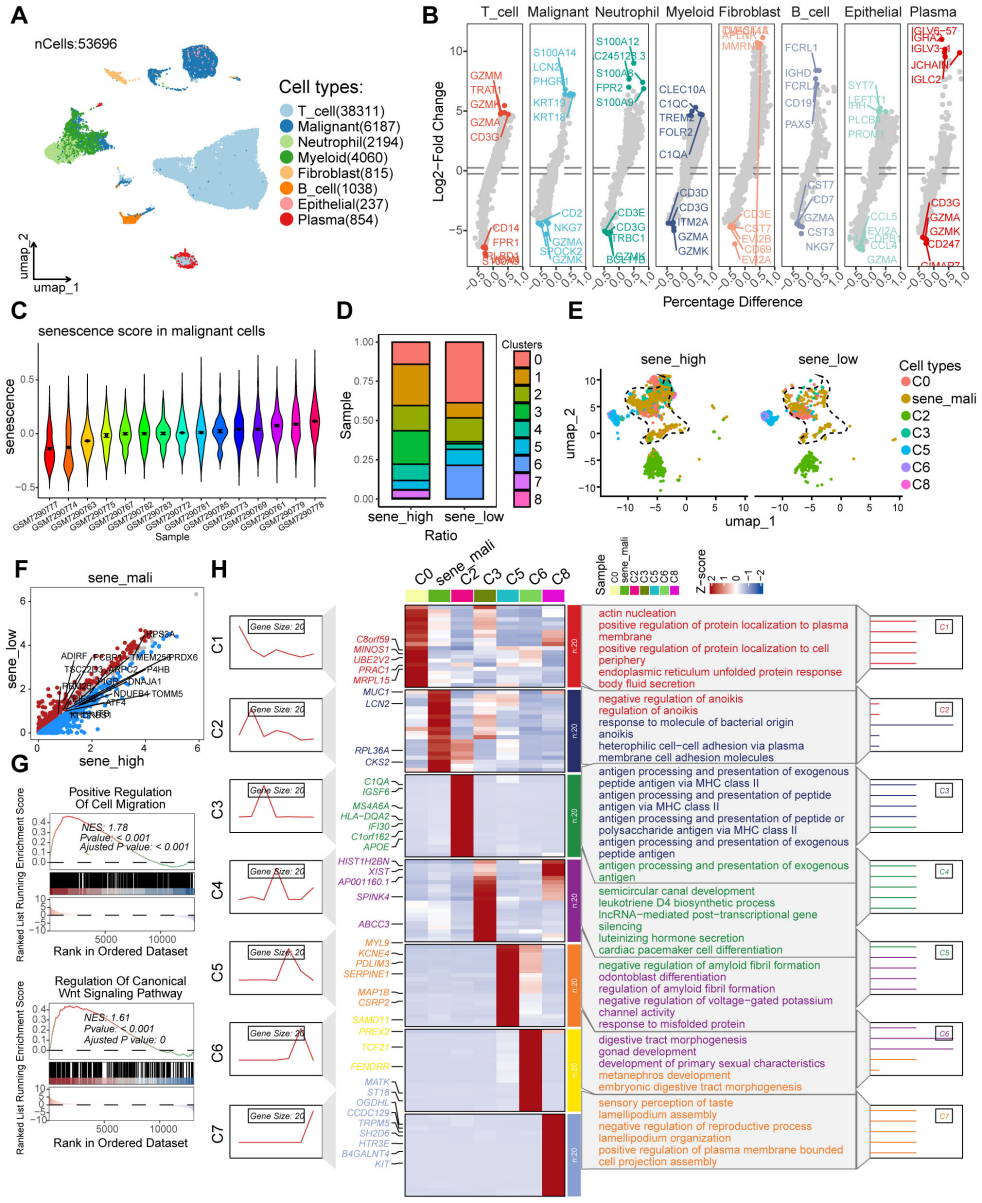
The scRNA-seq analysis to identify the secernent cell subsets. **(A)** UMAP plot showing the distribution of different cell types, including T_cell, Malignant, Neutrophil, Myeloid, Fibroblast, B_cell, Epithelial, and Plasma, in the dataset. **(B)** Violin plots displaying the Log2-Fold Change of gene expression across various cell types, highlighting differential expression patterns. **(C)** Violin plots illustrating the senescence score distribution in malignant cells across different samples. **(D)** Heatmap showing the ratio of different clusters (C0 to C8) in samples with high and low senescence scores. **(E)** UMAP plot showing the distribution of different cell types. **(F)** Volcano plots showing the differentially expressed genes in the malignant cell cluster of samples with low and high senescence scores. **(G)** GSEA plots for the enriched signaling pathways in the sample with high senescence scores, in comparison to low senescence scores. **(H)** Heatmap showing the expression of genes related to different clusters and their associated biological processes.

Following stringent quality control measures applied to a colorectal cancer scRNA-seq dataset (n = 53,696 cells) obtained from the GEO database, we excluded low-quality cells and retained high-confidence transcriptomes for subsequent analysis (**Supplementary Figures S1A, B**). Using cell-specific marker molecules, we classified the cells into eight major clusters: T lymphocytes, neoplastic cells, neutrophils, myeloid lineage cells, fibroblasts, B lymphocytes, epithelial cells, and plasma cells (**Figures 2A, B**). Subsequently, a senescence-associated gene set was extracted from the MSigDB. Utilizing this gene set, senescence scores were computed for the malignant cell cluster across various tumor samples. The samples were then stratified into high-senescence and low-senescence score groups, with the median senescence score serving as the threshold for categorization (**Figure 2C**). Among malignant subpopulations, we identified tumor cell clusters with significantly elevated senescence scores in the high-senescence score group as senescent tumor cell subpopulations. Notably, cluster 0 exhibited a marked increase in the high-senescence score group and was designated as the senescent tumor cell subpopulation (**Figures 2D, E**). To elucidate signaling pathways associated with the malignant cell subpopulation, we performed differential gene expression analysis between high- and low-senescence score groups, identifying 2,151 significantly differentially expressed genes (P < 0.05, |logFC| > 1) (**Figure 2F**). Gene Set Enrichment Analysis (GSEA) revealed prominent activation of pathways related to positive regulation of cell migration and canonical Wnt signaling in the high-senescence score subpopulation (**Figure 2G**). Subsequent pathway enrichment analysis using the GseaVis R package demonstrated significant heterogeneity among tumor cell subpopulations, including enrichment in endoplasmic reticulum unfolded protein response, heterophilic cell-cell adhesion, antigen processing and presentation, luteinizing hormone secretion, response to misfolded protein, embryonic digestive tract morphogenesis, and positive regulation of plasma membrane-bounded processes (**Figure 2H**). These results suggest that senescent tumor cell subpopulations in the colorectal cancer microenvironment contribute to diverse biological processes.

### Identification of genes associated with senescent tumor cell subpopulations using hdWGCNA

**Figure 3:**
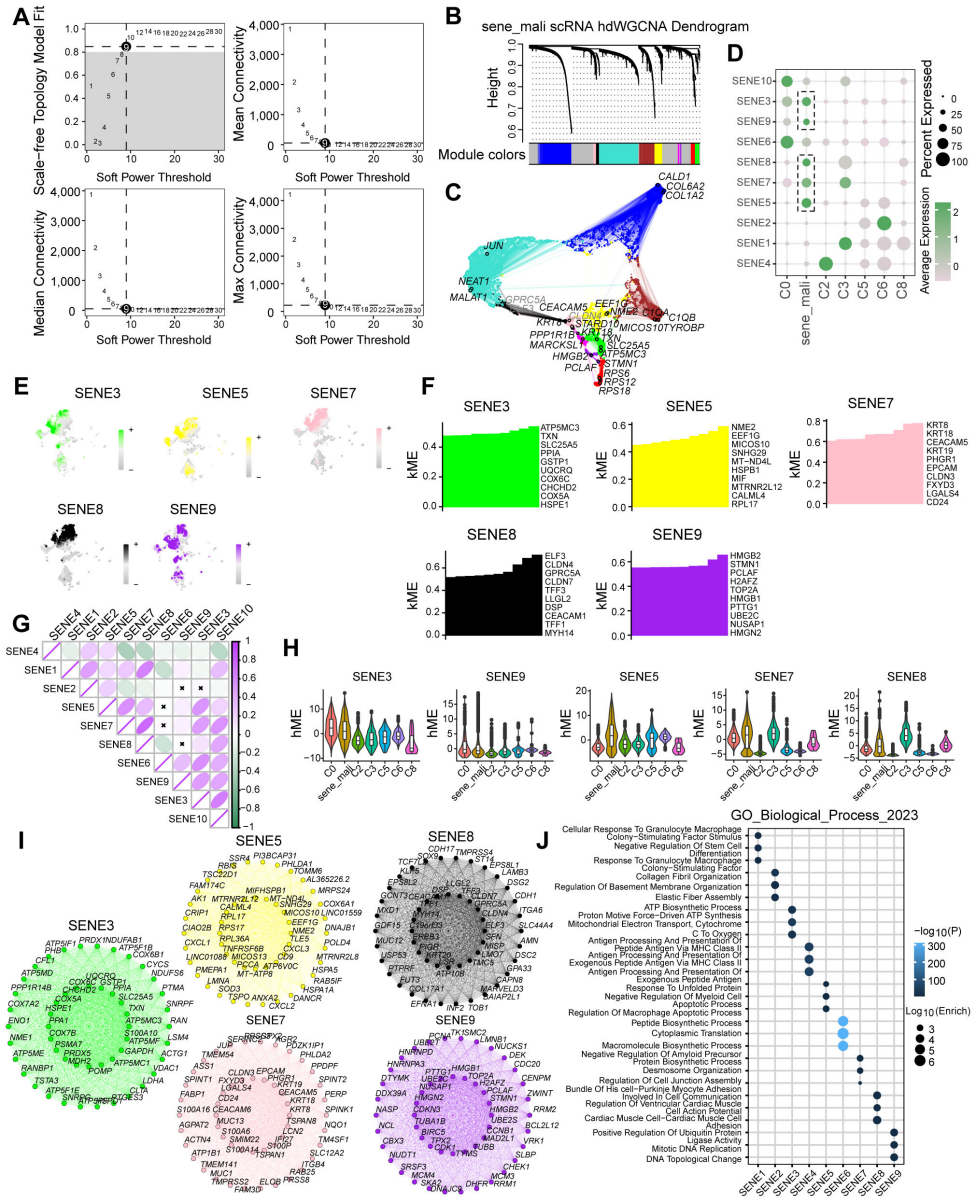
The identification of senescence related genes by hdWGCNA analysis. **(A)** Scale-free topology model selection aligns with minimal soft power threshold, ensuring network adherence to scale-free characteristics. **(B)** Co-expression network construction via optimal soft thresholding, followed by gene module clustering and module identification. **(C)** Metacell signatures and module-trait co-expression patterns derived using hdWGCNA. **(D)** Depiction of module activities across various macrophage clusters. **(E)** Gene scores calculated via UCell algorithm to quantify module gene expression levels. **(F)** kME calculations in co-expression network analysis to identify hub genes within modules. **(G)** Pearson correlation analysis assesses module intercorrelations. **(H)** Illustration of module activity across seven distinct clusters. **(I)** Identification of core genes within each module. **(J)** Highlighting enriched biological processes within individual modules.

To identify genes significantly associated with senescent tumor cell subpopulations, we applied hdWGCNA to tumor cell clusters identified through scRNA-seq analysis. After evaluating scale-free topology model fit and median connectivity, we established the optimal soft power threshold as 9 (**Figure 3A**). Using this threshold, we constructed a dendrogram of senescent tumor cell clusters and mapped the interactive gene networks across cell types (**Figures 3B, C**). We identified five modules-SENE3, SENE5, SENE7, SENE8, and SENE9-most strongly associated with senescent tumor cell clusters (**Figure 3D**). To characterize these modules, we performed hierarchical module eigengene (hME) analysis. The hME, which represents the first principal component of gene expression within a module, summarizes the module’s expression profile. This measure quantifies overall expression patterns and enables assessment of module-module correlations as well as associations with clinical traits (**Figures 3E, F**). Significant inter-module correlations were further illustrated in a pie chart. Strikingly, senescent tumor cell subpopulations displayed elevated module eigengene (kME) values relative to other tumor cell subsets (**Figures 3G, H**). To determine the biological relevance of these modules, we conducted network interaction analysis of module genes followed by Gene Ontology (GO) enrichment analysis, which identified key biological processes associated with each module (**Figures 3I, J**). Collectively, hdWGCNA provided a systematic framework to identify senescence-associated modules and genes across tumor cell subpopulations, establishing a basis for subsequent functional and mechanistic studies.

### Construction of a prognostic model using machine learning to identify colorectal cancer-specific signatures

**Figure 4:**
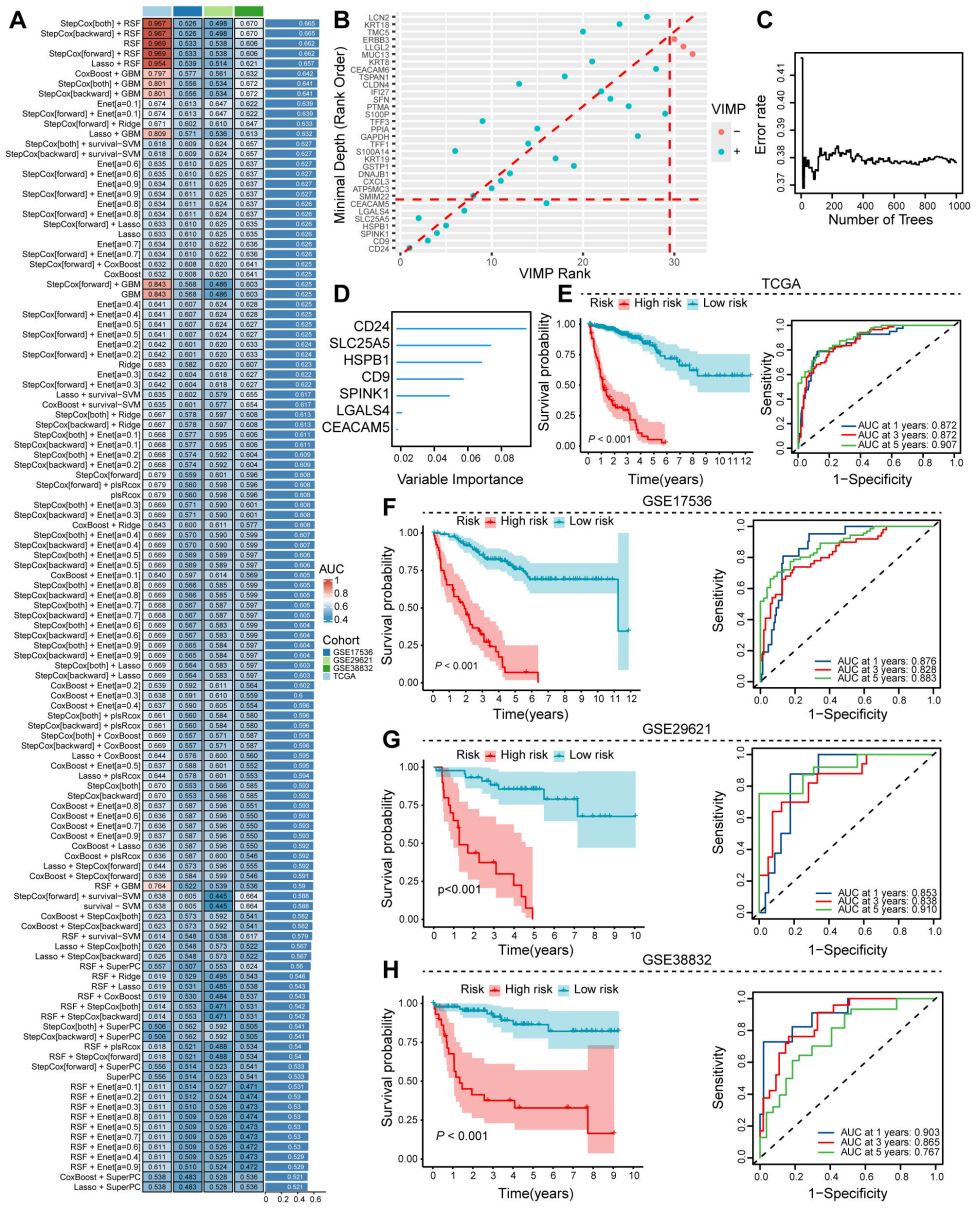
Machine learning approaches to discern prognostic senescence-associated gene signatures. **(A)** Ensemble machine learning algorithms employed for optimal prognostic model determination. **(B–D)** RSF analysis utilized to pinpoint critical genes associated with prognosis. **(E)** TCGA training dataset assesses the prognostic and predictive capabilities of the identified senescence gene set. **(F–H)** Validation of the senescence gene set’s prognostic and predictive performance on colon cancer sequencing data from the GEO database.

Using hdWGCNA analysis, we identified a robust gene set comprising 38 genes strongly associated with tumor cell senescence. To construct a prognostic model, we evaluated multiple machine learning algorithms and determined that StepCox+RSF exhibited the optimal predictive performance based on AUC values (**Figure 4A**). Using Random Survival Forest (RSF) analysis with importance scoring, we identified seven key prognostic genes (CD24, SLC25A5, HSPB1, CD9, SPIMK1, LGALS4, and CEACAM5; **Figures 4B–D**), which were subsequently designated as the Colorectal cancer Survival Signature (CSS). In the TCGA-COAD cohort, the CSS effectively stratified patients by survival outcomes, demonstrating that higher CSS scores were significantly associated with worse prognosis. Time-dependent ROC analysis reinforced the predictive capacity of CSS, showing AUC values of 0.872 (1-year), 0.872 (3-year), and 0.907 (5-year) (**Figure 4E**). External validation using independent GEO datasets confirmed the consistent prognostic performance of CSS across different cohorts (**Figures 3F–H**). These results establish CSS as a robust prognostic indicator for identifying high-risk colorectal cancer patients, with potential clinical applications for risk stratification and personalized treatment decisions.

### Heterogeneity of senescent tumor cells and their association with immunosuppressive tumor microenvironment

**Figure 5:**
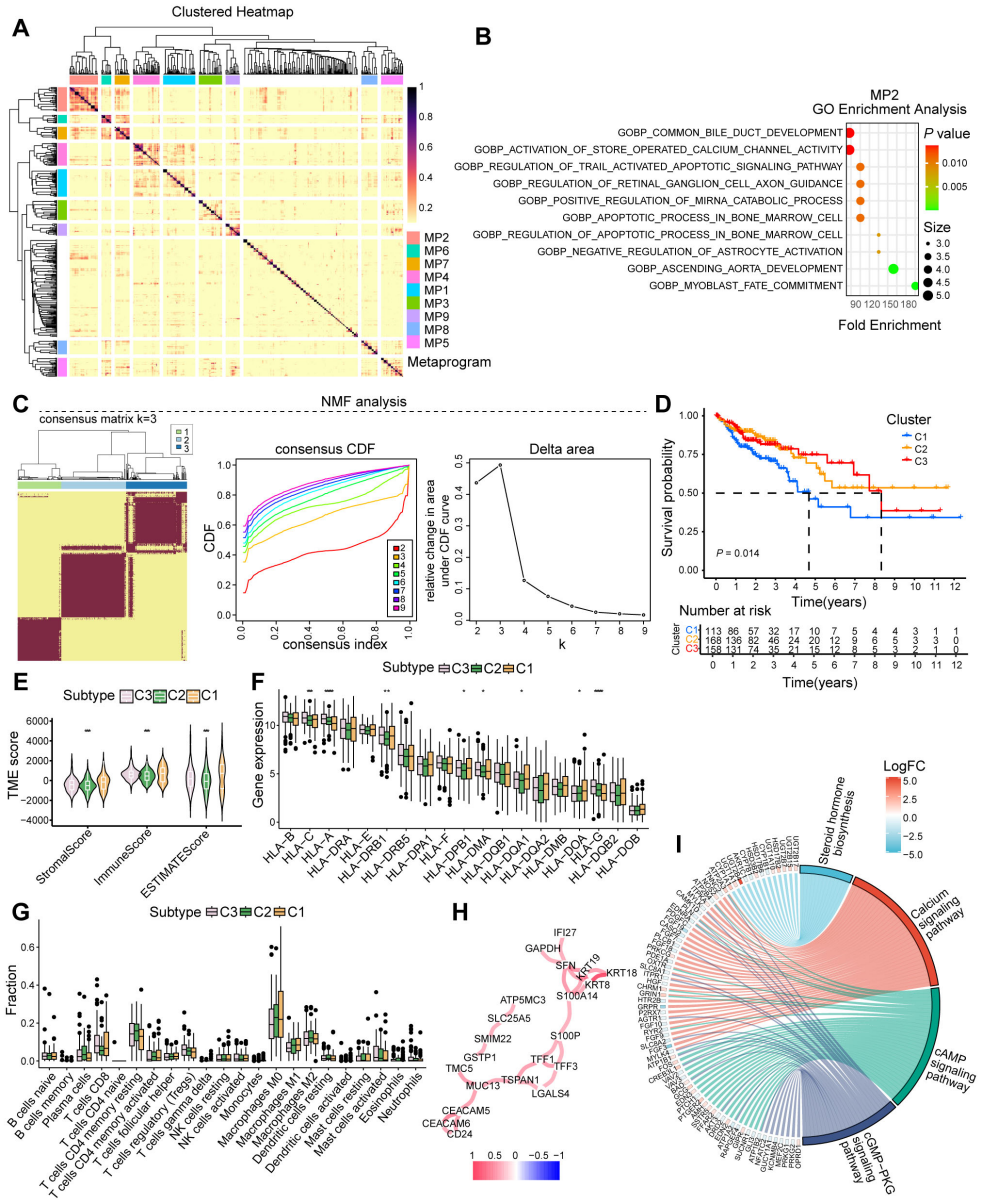
Analysis of cellular senescence heterogeneity and immunophenotyping. **(A)** Heatmap illustrating the heterogeneity among senescent cells. **(B)** GO analysis depicting enriched biological processes in MP2. **(C)** Typing modules from NMF analysis and determination of the optimal K value. **(D)** Prognosis of patients across various clusters. **(E)** Microenvironment scoring analysis. **(F)** Expression analysis of immune-related genes. **(G)** Analysis of immune cell infiltration. **(H)** Interaction network of core genes. **(I)** Enriched signaling pathways or biological processes of differentially expressed genes. *, *P* < 0.05, **, *P* < 0.01, ***, *P* < 0.001, *****, *P* < 0.00001.

To characterize the role of senescent tumor cells, we employed Non-negative Matrix Factorization (NMF) on scRNA-seq data, uncovering substantial heterogeneity across senescent tumor cell subpopulations (**Figure 5A**). GO enrichment analysis of metaprogram 2 (MP2) highlighted biological processes such as common bile duct development and activation of store-operated calcium channel activity, both of which exhibited significant positive correlations with senescent cell heterogeneity (**Figure 5B**). Leveraging the CSS, we applied NMF to stratify patients from the TCGA-COAD cohort into three distinct clusters (**Figure 5C**). Comparative analysis revealed significant prognostic differences, with cluster 1 patients exhibiting significantly worse survival outcomes than cluster 3 (**Figure 5D**). The poor prognosis in cluster 1 was associated with a less immunogenic tumor microenvironment, characterized by lower TME scores, increased infiltration of immunosuppressive M0 macrophages, and downregulation of key antigen-presenting molecules including HLA-G (**Figures 5E–G**). To further delineate the functional implications of CSS, we divided COAD patients into high- and low-CSS groups based on median expression. Differential gene interaction network analysis identified CSS as significantly enriched in pathways including steroid hormone biosynthesis, calcium signaling pathway, cAMP signaling pathway, and cGMP-PKG signaling pathways (**Figures 5H, I**). Collectively, these findings underscore the heterogeneity of senescent tumor cells and association with poor prognosis and an immunosuppressive TME, providing insights into the mechanistic underpinnings of CSS in colorectal cancer progression.

### CSS expression is negatively correlated with low T cell infiltration and antitumor function

**Figure 6:**
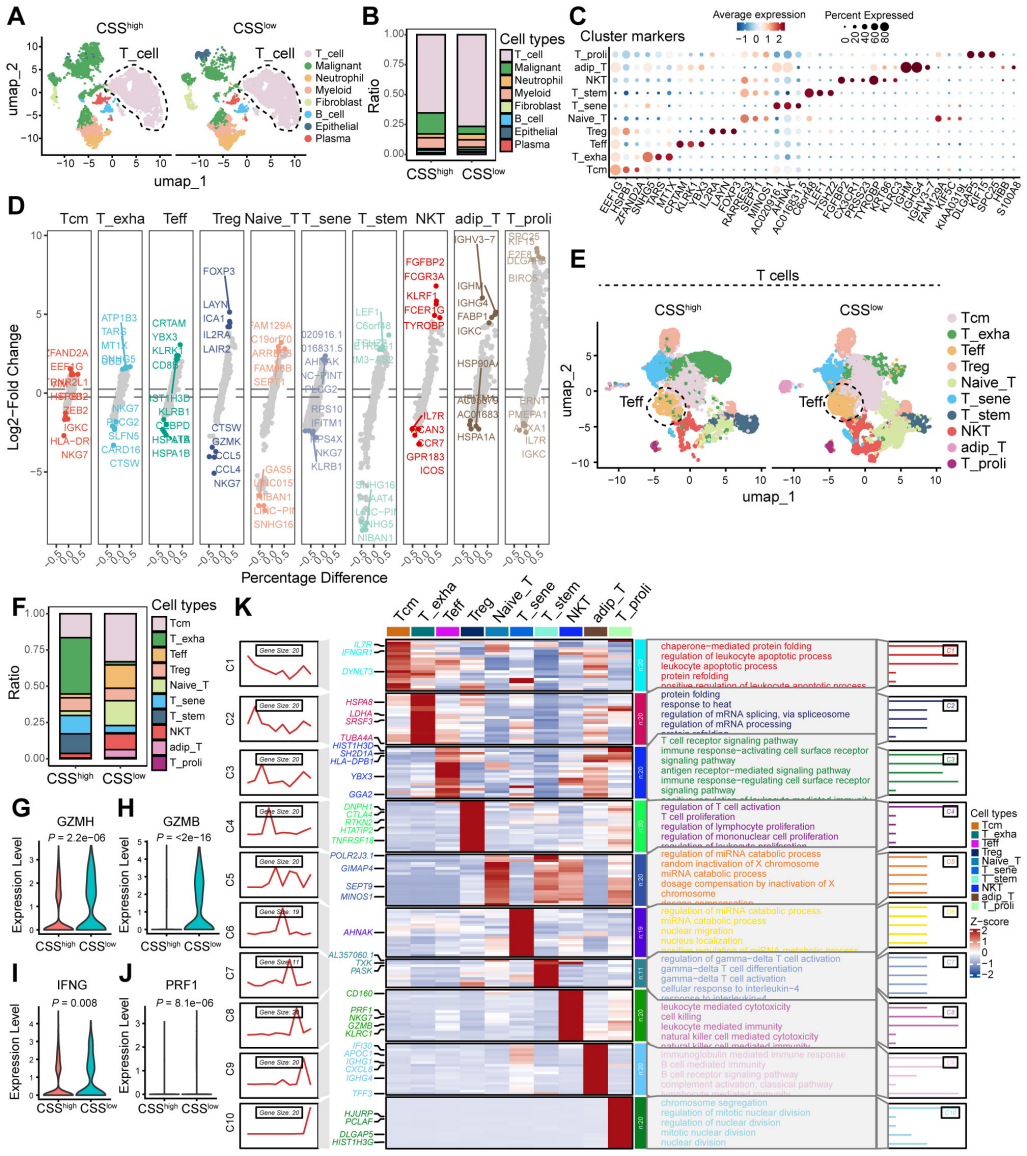
The effect of CCS in the TME of colorectal cancer. **(A)** UMAP plot showing the cell clusters between CSS^low^ and CSS^high^. **(B)** Bar graphs showing the ratio of different cell types in CSS^low^ and CSS^high^ clusters. **(C)** Scatter plot of average expression for cluster markers, with corresponding cell types indicated. **(D)** Volcano plot showing the Log2-Fold Change of differentially expressed genes between various T cell subsets. **(E)** UMAP plot illustrating the distribution of T cell subsets. **(F)** Bar graphs showing the ratio of different T cell subsets between CSS^low^ and CSS^high^ clusters. **(G)** Heatmap displaying the percentage difference in gene expression across various T cell subsets. **(H–K)** Violin plots depicting the expression levels of GZMH, GZMB, IFNG, and PRF1 in Teff clusters of samples with CSS^low^ and CSS^high^.

To investigate the influence of CSS on the TME, we conducted an analysis of scRNA-seq data from colorectal cancer. Our analysis revealed a markedly higher proportion of T cells in CSS-low tumors compared to CSS-high tumors (**Figures 6A, B**). Following the isolation of T cell populations, we employed dimensionality reduction and clustering techniques to comprehensively annotate T cell clusters according to canonical marker expression. Within the T cell compartment, we identified 10 distinct subpopulations, including central memory T cells (Tcm), effector T cells (Teff), exhausted T cells (T_exha), regulatory T cells (Treg), naïve T cells (Naïve_T), senescent T cells (T_sene), stem-like T cells (T_stem), natural killer T cells (NKT), adipose-resident T cells (adip_T), and proliferating T cells (T_proli). Notably, CSS-low tumors exhibited a significant enrichment of Teff cells (**Figures 6C–E**). To characterize the functional activation of Teff cells, we analyzed their effector molecule profiles, revealing significant upregulation of cytotoxic mediators (GZMH, GZMB, IFNG, and PRF1) in CSS-low tumors (**Figures 6F–J**). GSEA analysis of Teff-specific signatures showed strong enrichment of T cell-mediated immune response pathways, particularly those associated with T cell receptor signaling, immune response activation through surface receptor signaling, antigen recognition-mediated signaling, and immunomodulatory surface receptor signaling (**Figure 6K**). These findings collectively indicate that reduced CSS expression promotes T cell infiltration and potentiates their antitumor functionality within the TME, highlighting its role in shaping immunologically active tumor microenvironments.

### CSS inhibits CD8^+^ T cell activation by suppressing tumor antigen presentation signaling

**Figure 7:**
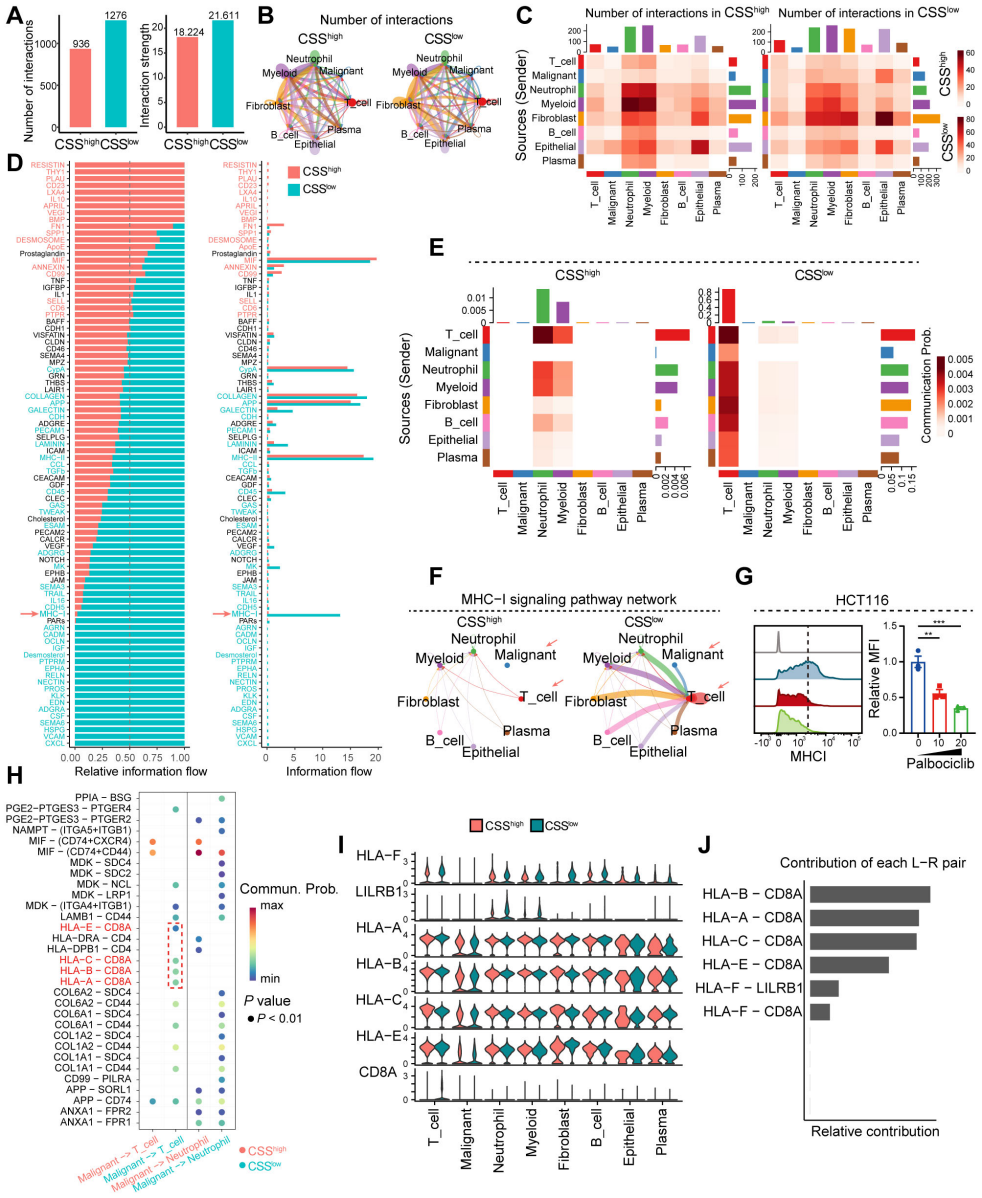
Analysis of cell-cell interactions and signaling pathways in the tumor microenvironment. **(A)** Bar graph showing the number of interactions and interaction strength between different cell types. **(B)** Network diagram illustrating the number of interactions among various cell types. **(C)** Heatmaps comparing the number of interactions and communication probabilities between cell types in CSS^low^ and CSS^high^ states. **(D)** Bar graph depicting the relative information flow of ligand-receptor interactions in CSS^low^ and CSS^high^ states. **(E)** Heatmaps showing MHC-I signaling in CSS^low^ and CSS^high^ states across different cell types and sources. **(F)** Network diagram of the MHC-I signaling pathway, indicating interactions between various cell types in CSS^low^ and CSS^high^ states. **(G)** Flow cytometry analysis showing the MHCI expression in HCT116 cells treated with distinct concentration of palbociclib. **(H)** Dot plot showing the ligand-receptor interactions. **(I)** Violin plot depicting the ligand-receptor interactions in distinct cell clusters between CSS^low^ and CSS^high^ states. **(J)** Bar graph illustrating the contribution of each L-R (ligand-receptor) pair to the overall signaling. **, *P* < 0.01, ***, *P* < 0.001.

To uncover the molecular underpinnings through which CSS modulate T cell-driven antitumor immune responses, we conducted cell-cell communication analysis utilizing scRNA-seq data derived from colorectal cancer samples. The analysis revealed that reduced CSS expression correlates with increased frequency and intensity of intercellular interactions within the tumor microenvironment (**Figure 7A**). Specifically, diminished CSS expression was found to significantly enhance the interaction between tumor cells and T cells (**Figures 7B, C**). Signal flow analysis further demonstrated that suppression of CSS robustly augments MHC-I signaling, a critical pathway for CD8^+^ T cell activation. Enhanced MHC-I signaling in tumor cells promotes the activation and functional maturation of CD8^+^ T cells, thereby facilitating their cytotoxic potential against tumor cells (**Figures 7D–F**). *In vitro*, palbociclib-induced cellular senescence was modeled in HCT116 cells. Comparative analysis revealed a significant downregulation of MHC class I expression in senescent cells relative to control groups (**Figure 7G**). To identify the molecular determinants underlying this process, we conducted ligand-receptor interaction analysis, which revealed that low CSS expression induces the upregulation of HLA-A/B/C/E ligands in tumor cells. These ligands engage with CD8A on T cells, driving their activation and potentiating antitumor responses (**Figure 7H**). Moreover, in the CSS-low tumor microenvironment, CD8A expression on T cells was substantially elevated, and the HLA-A/B/C-E-CD8A interaction emerged as the most pronounced ligand-receptor axis (**Figures 7I, J**). Collectively, these results imply that reduced CSS expression potentiates antitumor immunity by upregulating MHC-I signaling in tumor cells, leading to enhanced activation and functional efficacy of CD8^+^ T cells.

### High CSS expression is negatively correlated with immunotherapy response in cancer patients

**Figure 8:**
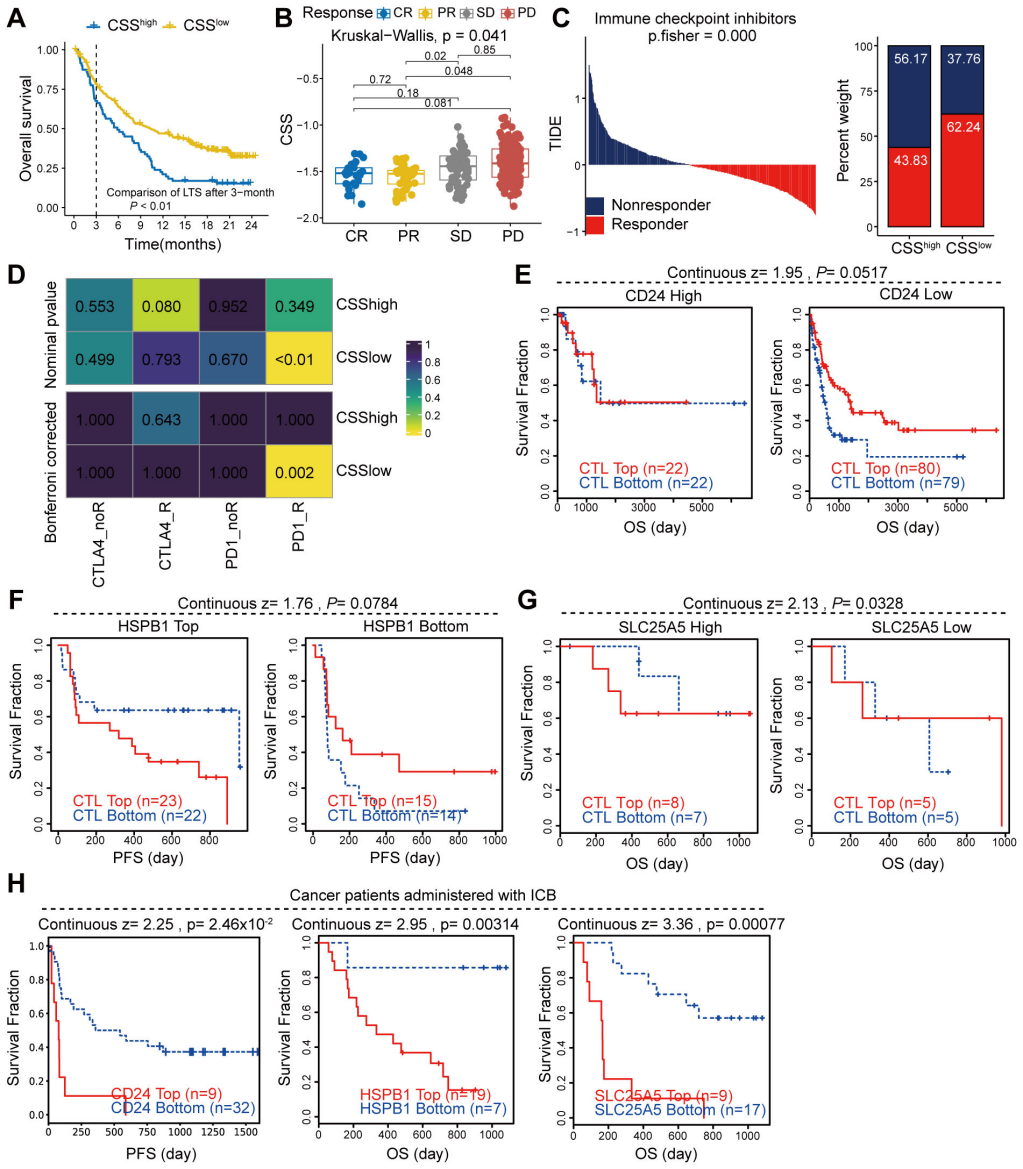
The correlation between CCS expression and immunotherapy response. **(A, B)** Analysis of the immunotherapy cohort IMvigor210 to assess the influence of CSS on patient prognosis and response to immunotherapy. **(C)** Utilization of the TIDE database to evaluate the effect of CSS on immune responses in patients with colon cancer. **(D)** Heatmap demonstrating the correlation between CSS and response to immunotherapy. **(E, F)** Examination of the impact of key gene expression within CSS on patient prognosis mediated by CTL (cytotoxic T lymphocytes). **(H)** Survival of cancer patients treated with ICB, stratified by gene expression.

To evaluate the association between CSS and clinical outcomes in immunotherapy-treated cancer patients, we conducted comprehensive analyses using the IMvigor210 cohort and the TIDE database. Our findings revealed that elevated CSS expression correlated markedly with adverse prognosis in the IMvigor210 cohort (**Figure 8A**). Our analysis revealed significantly higher CSS expression levels in immunotherapy non-responders (SD/PD) compared to responders (**Figure 8B**). Integration of TCGA colorectal cancer sequencing data with TIDE immunotherapy datasets demonstrated significantly lower response rates among patients with elevated CSS expression. Notably, colorectal cancer patients receiving anti-PD1 therapy exhibited superior treatment responses when CSS expression was low (**Figures 8C, D**). Validation using melanoma data from TIDE revealed that key CSS-associated molecules (CD24, HSPB1, SLC25A5) profoundly influenced immunotherapy outcomes. Patients demonstrating strong CTL infiltration showed improved clinical responses when these molecules were downregulated, whereas their overexpression negated the therapeutic benefit (**Figures 8E–G**). These findings collectively suggest that high CSS expression may attenuate CTL-mediated anti-tumor immunity, thereby compromising immunotherapy efficacy. Moreover, melanoma patients receiving immune checkpoint blockade (ICB) therapy with high expression of CD24, HSPB1, and SLC25A5 also had a poor prognosis (**Figure 8H**). In summary, our findings indicate that elevated expression of CSS is strongly associated with diminished responsiveness to immunotherapy and a heightened risk of poor prognosis in patients receiving ICB treatment.

### Blocking CD24 inhibited malignant phenotypes of tumor cells

**Figure 9:**
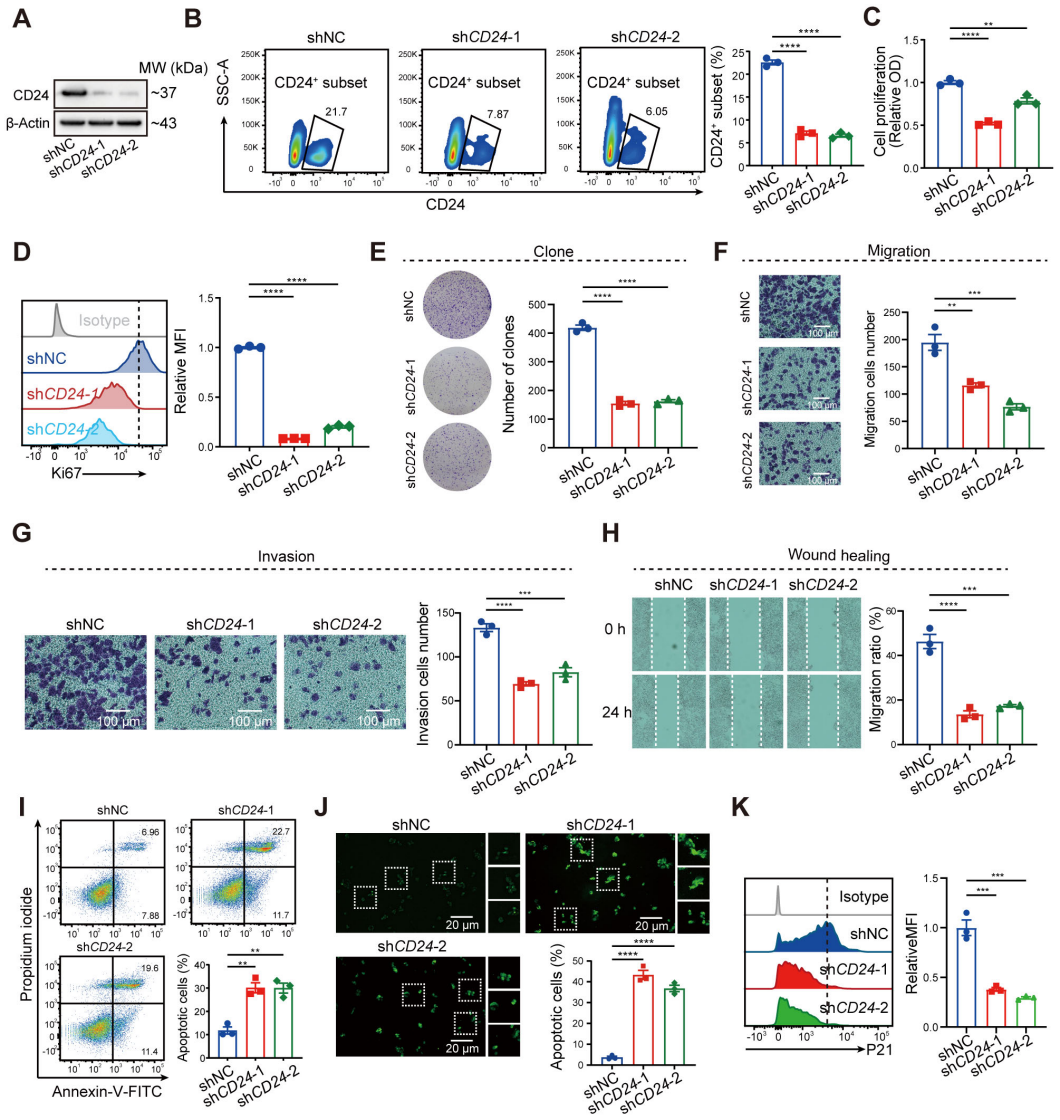
Blockade of CD24 significantly inhibited malignant phenotypes of tumor cells. **(A)** Western blot analysis representing the CD24 expression. **(B)** Flow cytometry analysis showing the CD24^+^ cells ratio. **(C)** CCK-8 assay showing the proliferation of HCT116 cells. **(D)** Flow cytometry analysis illustrating the Ki67 expression. **(E, F)** Cell proliferation **(E)** and migration **(F)** of HCT116 cells. **(G, H)** Analysis of invasion **(G)** and wound healing assay **(H)**. **(I, J)** Flow cytometry **(I)** and TUNEL staining **(J)** showing the ratio of cell apoptosis. **(K)** Flow cytometry analysis showing the P21 expression in shNC and shCD24 HCT116 cells. **, *P* < 0.01, ***, *P* < 0.001, ****, *P* < 0.0001.

In our investigation of senescence-associated signatures in colorectal cancer, CD24 emerged as the most critically prioritized gene, exhibiting the highest importance score (**Figure 4D**). To dissect its functional contribution to malignant progression, we engineered CD24-knockdown HCT116 cell lines via shRNA-mediated silencing, confirmed by significant CD24 protein reduction through western blot and flow cytometric analyses (**Figures 9A, B**). Subsequent functional characterization revealed multifaceted oncogenic roles of CD24: its depletion markedly suppressed proliferative capacity, as evidenced by diminished CCK8 absorbance and reduced KI67-positive subpopulations (**Figures 9C, D**), while concurrently impairing clonogenicity (**Figure 9E**) and migration/invasion potentials in transwell, wound healing, and Matrigel assays (**Figures 9F–H**). Furthermore, CD24 deficiency substantially elevated apoptotic rates, validated by Annexin V/PI staining and TUNEL assays (**Figures 9I, J**). Furthermore, CD24 knockdown in tumor cells markedly suppressed the expression of the senescence marker P21 compared with control cells (**Figure 9K**). Collectively, these findings identify CD24 as a pivotal regulator within the CRC senescence network, coordinating tumor progression through concurrent enhancement of proliferative capacity, metastatic potential, and apoptosis resistance.

### Afatinib targeting CSS to promote growth inhibition of senescent tumor cells

**Figure 10:**
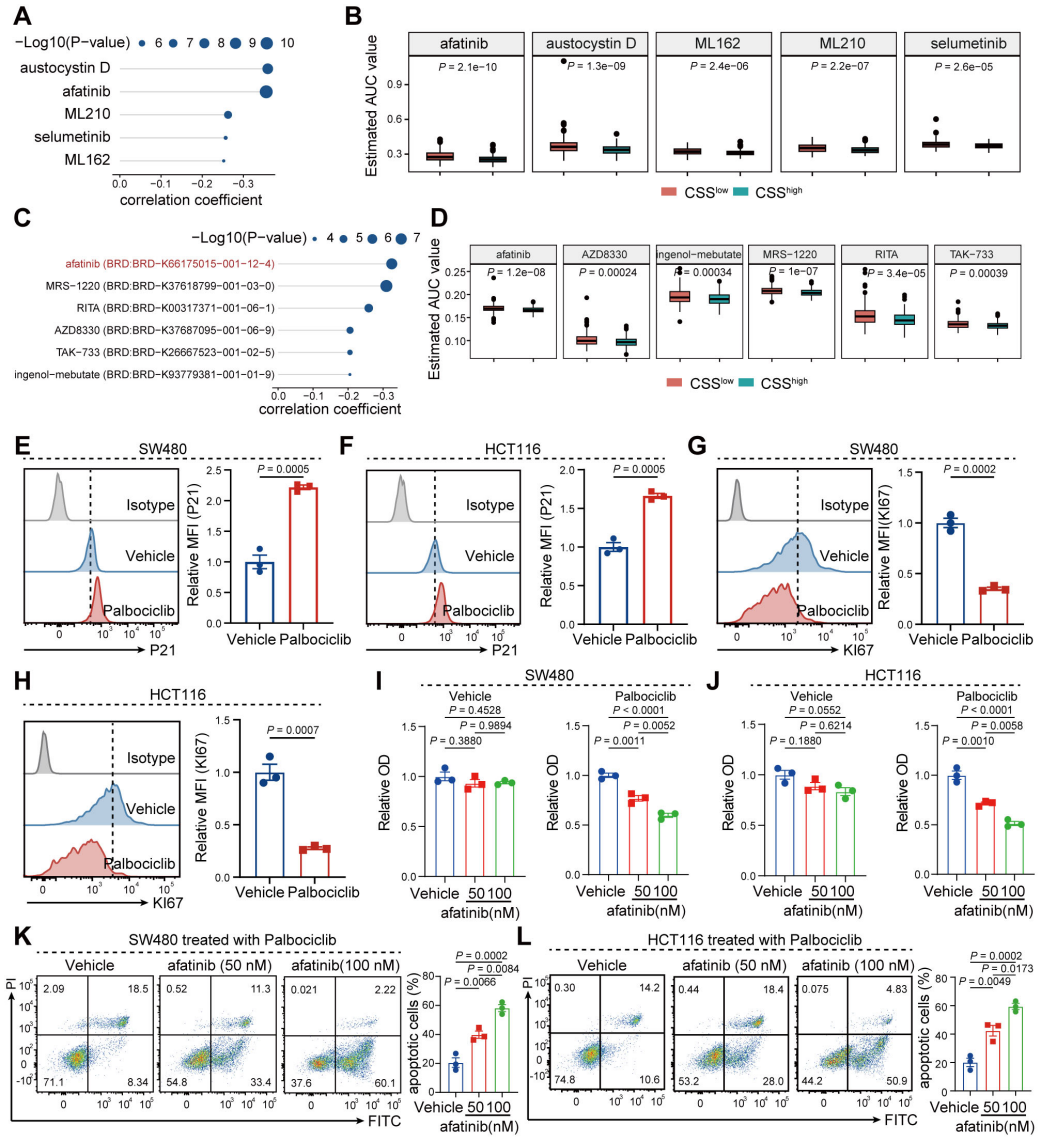
Afatinib promotes apoptosis in senescent tumor cells by targeting CSS. **(A)** Correlation analysis showing the correlation between CSS and the various compounds in the CTRP database. **(B)** Box plots representing the estimated AUC (Area Under Curve) values. **(C)** Scatter plot depicting the correlation coefficient between CSS and compounds in the PRISM database. **(D)** Box plots illustrating the estimated AUC values in response to various compounds in the PRISM database. **(E, F)** Flow cytometry histograms comparing the relative MFI (Mean Fluorescence Intensity) of P21 and KI67 in SW480 and HCT116 cells treated with vehicle or palbociclib. **(G)** Flow cytometry histograms showing the relative MFI of KI67 in SW480 cells treated with vehicle or palbociclib. **(H)** Flow cytometry histograms comparing the relative MFI of KI67 in HCT116 cells treated with vehicle or palbociclib. **(I, J)** Bar graphs representing relative OD (Optical Density) values in SW480 **(I)** and HCT116 **(J)** cells treated with palbociclib and varying concentrations of afatinib. **(K, L)** Flow cytometry histograms showing the proportion of apoptotic cells in SW480 **(K)** and HCT116 **(L)** cells treated with palbociclib and different concentrations of afatinib.

To identify therapeutic agents targeting the CSS in COAD, we integrated transcriptomic profiles from the TCGA-COAD dataset with drug sensitivity data from the CTRP and PRISM databases. This multi-database computational approach prioritized afatinib as a candidate with potential CSS-targeting activity (**Figures 10A–D**). We validated the effects on senescent tumor cells using palbociclib-induced senescence models in HCT116 and SW480 colorectal cancer cell lines. Flow cytometry confirmed efficient senescence induction, as evidenced by increased P21 expression (senescence marker) and decreased KI67 levels (proliferation marker) (**Figures 10E–H**). While afatinib showed negligible cytotoxicity in non-senescent cells at 50–100 nM concentrations, it displayed significant dose-dependent anti-proliferative and pro-apoptotic effects selectively in senescent tumor cells (**Figures 10I–L**). This selective vulnerability of senescent cells to afatinib underscores the therapeutic potential of CSS-targeted strategies in modulating the TME. Collectively, these findings position afatinib as a promising senolytic agent capable of selectively eliminating therapy-induced senescent tumor cells, thereby hindering tumor progression.

## Discussion

Colorectal cancer continues to impose a significant global health burden, with limited effective therapeutic options for many patients (8, 26). This study identifies CSS, specifically centered on CD24, as a significant predictor of patient prognosis and response to immunotherapy in CRC. This finding provides a novel framework for deciphering the complex mechanisms underpinning CRC progression and therapeutic resistance. Our data robustly demonstrate that elevated CSS levels correlate with diminished T cell infiltration and a functionally impaired CD8^+^ T cell compartment within the TME. This association strongly suggests a pivotal role for CSS in facilitating immune evasion and driving tumor progression in CRC. Mechanistically, CSS attenuates the cytotoxic potential of CD8^+^ T cells by suppressing tumor cell antigen presentation via the MHC-I signaling pathway. These observations collectively highlight the therapeutic potential of targeting CSS to enhance the efficacy of immunotherapeutic strategies for CRC patients. The identification of CSS as both a prognostic and predictive biomarker carries considerable clinical significance. It enables the stratification of CRC patients into distinct risk categories, thereby informing tailored therapeutic approaches. For instance, patients exhibiting high CSS expression may derive benefit from interventions targeting the MHC-I pathway or modalities designed to augment T cell infiltration and function within the TME. Furthermore, a prognostic model incorporating CSS could facilitate the monitoring of disease progression and prediction of treatment outcomes, paving the way for more personalized and effective clinical management.

Beyond its established roles in immune modulation, CD24 exhibits multifaceted functions relevant to cancer biology. In the context of oncogenesis, CD24 is frequently overexpressed and contributes critically to tumor progression by promoting cancer cell proliferation, migration, invasion, metastasis, and the maintenance of stem-like properties (27, 28). Notably, CD24 has also been implicated in modulating cellular senescence, a state of stable growth arrest often acting as a tumor-suppressive barrier. While CD24 expression can be induced in some senescent contexts, it paradoxically also plays a role in enabling certain cancer cells to evade senescence or modulate the SASP, thereby influencing the tumor microenvironment (29). Crucially, our study establishes a direct link between CD24 and the induction of a senescence-like phenotype in tumor cells. We demonstrate that elevated CD24 expression correlates with and functionally contributes to cellular senescence within CRC, a novel finding that expands our understanding of CD24’s oncogenic mechanisms. Consequently, CD24 emerges not only as a biomarker but also as a promising direct therapeutic target.

Our investigation identified afatinib as a pharmacological agent capable of effectively targeting and inhibiting CSS, suggesting its potential utility in CRC treatment regimens. Afatinib’s documented capacity to inhibit senescent cell proliferation and induce apoptosis provides a compelling mechanistic rationale for its application in targeting CSS-related pathways, particularly given our novel finding linking CD24 to senescence induction in CRC. Future research priorities include evaluating the efficacy of afatinib and analogous agents in robust preclinical CRC models, both as monotherapies and in rational combination strategies with established immunotherapies. Furthermore, the interplay between CSS-driven senescence, immune evasion, and therapeutic response warrants deeper mechanistic exploration. Despite these promising insights, limitations of the current study must be acknowledged. The sample size employed for CSS identification and initial validation was relatively modest, necessitating confirmation in larger, independent patient cohorts. Additionally, the precise molecular mechanisms by which CSS orchestrates immunotherapy resistance and influences patient prognosis are likely more intricate than currently delineated, demanding further comprehensive investigation.

In conclusion, this study establishes CSS as a critical regulator of immune evasion, tumor progression, and cellular senescence in CRC, functioning as a valuable prognostic/predictive biomarker and a viable therapeutic target. The identification of afatinib as a CSS inhibitor offers a tangible translational avenue. These findings collectively lay a robust foundation for future research aimed at developing innovative strategies to improve the management and outcomes of CRC patients.

## Conclusion

In summary, our findings establish CSS as a dual-purpose prognostic indicator for predicting both clinical outcomes and immunotherapy resistance in colorectal cancer patients. Mechanistic investigations reveal that CSS promotes immunosuppression primarily by impairing CD8^+^ T-cell infiltration and cytotoxic function, mediated through downregulation of MHC class I antigen presentation. Notably, pharmacological intervention using afatinib effectively inhibited CSS activity, exhibiting robust antitumor effects through targeted elimination of senescent CRC cells via apoptosis induction and proliferation blockade, thereby offering a viable therapeutic approach to overcome senescence-mediated immune escape.

## Data Availability

The original contributions presented in the study are included in the Article/Supplementary Material. Further inquiries can be directed to the corresponding author. The data presented in the study are deposited in the Gene Expression Omnibus repository (http://www.ncbi.nlm.nih.gov/geo/), accession number: GSE231559, GSE38832, GSE17536, and GSE29621.
