# Supplementary material for: Cancer-specific senescence signature promotes malignant phenotypes and immunotherapy resistance in colorectal cancer
Source: Front Immunol. 2025 Jul 24;16:1603787. doi: 10.3389/fimmu.2025.1603787 (PMC12328160; doi:10.3389/fimmu.2025.1603787)
Supplement: Supplementary file 1 [file DataSheet1.docx]

**Supplementary Figures for** **Cancer-specific senescence signature promotes malignant phenotypes and immunotherapy resistance in colorectal cancer**

**Wei Wang^1,2^, Fengyu Ling^1,2^, Dong Huang^3^, Guomin Luo^1,2^, Bixia Duan^1,2^**

**Supplementary Figure 1**

**
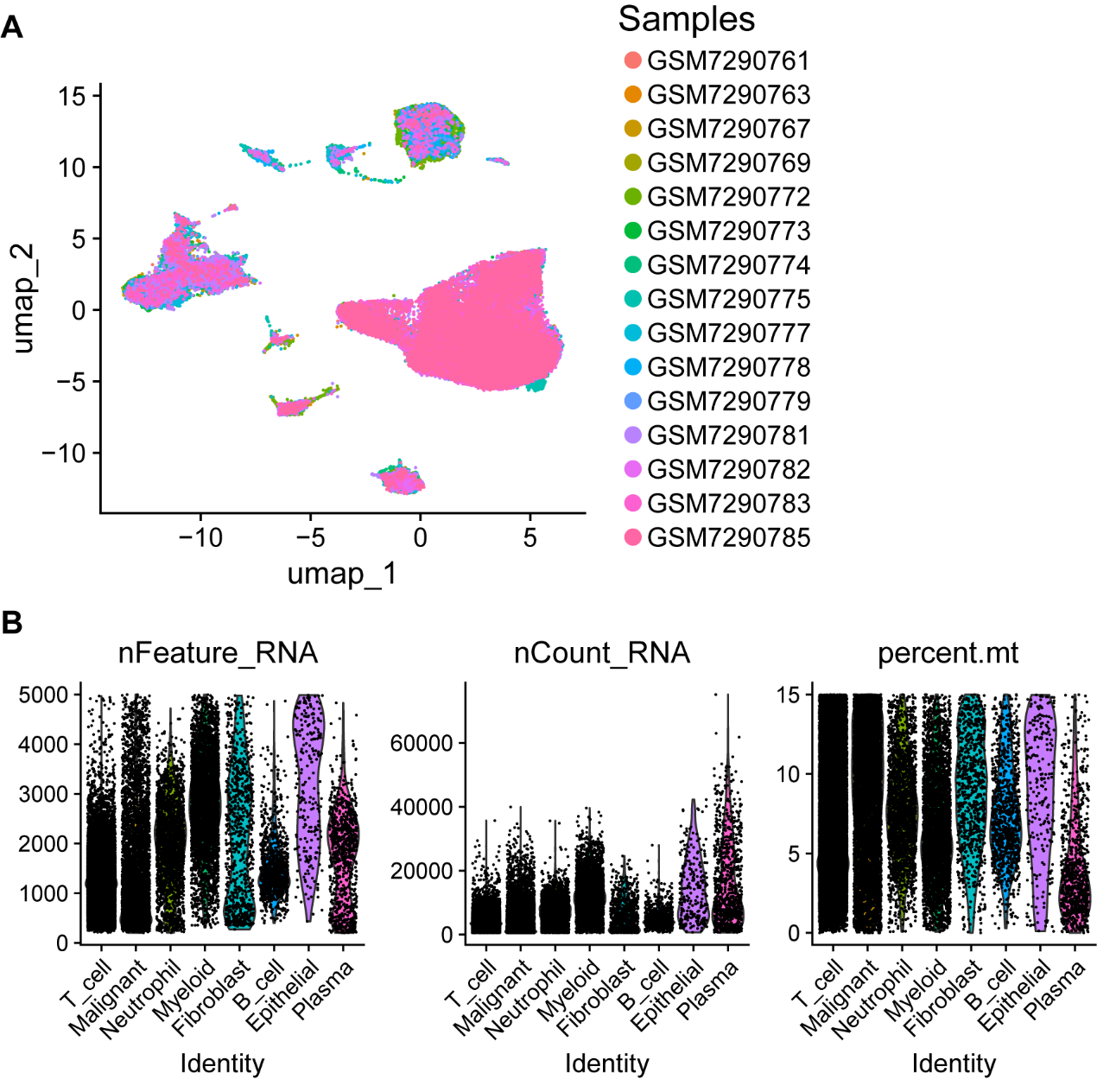
**

**Figure S1. Quality assessment of single-cell RNA sequencing data.**

**(A**) UMAP projection reveals the cellular distribution across samples, indicating the presence of batch effects. (**B**) Violin plots display key quality metrics, including the number of detected genes (nFeature_RNA), total UMI counts (nCount_RNA), and mitochondrial gene percentage (percent.mt), demonstrating the rigor of data filtering.
